# Dapagliflozin Improves High‐Fat Diet‐Induced Cognitive Impairment in Female Mice

**DOI:** 10.1002/brb3.70361

**Published:** 2025-02-19

**Authors:** Xiaolin Chen, Mingxia Fan, Zhuoni Xiao, Xiaoxing Xiong

**Affiliations:** ^1^ Department of Endocrinology Renmin Hospital of Wuhan University Wuhan China; ^2^ Center for Animal Experiment Renmin Hospital of Wuhan University Wuhan China; ^3^ Reproductive Medical Center Renmin Hospital of Wuhan University Wuhan China; ^4^ Department of Neurosurgery Renmin Hospital of Wuhan University Wuhan China

**Keywords:** cognitive impairment, dapagliflozin, high‐fat diet, hippocampus

## Abstract

**Background:**

High fat consumption is a known risk factor for the development of type 2 diabetes mellitus (T2DM) and Alzheimer's disease (AD). Sodium‐glucose cotransporter 2 inhibitors (SGLT2is) have been found to possess anti‐inflammatory and neuroprotective properties. However, the cognitive effects and mechanisms of SGLT2is on female mice fed with a high‐fat diet remain unknown.

**Objective:**

This study aimed to investigate the impacts of dapagliflozin on metabolism, cognition, neuroinflammation, insulin resistance, and microglial activation in female mice fed a HFD.

**Methods:**

Dapagliflozin (1 mg/kg) was administered to HFD‐fed mice for 24 weeks. Body weight, glucose tolerance, and insulin resistance were assessed. Additionally, all mice were subjected to the Morris water maze (MWM) and one‐trial Y‐maze tests. The levels of metabolic hormones and cytokines were analyzed using ELISA kits. The levels of phosphorylated tau (p‐tau) protein in the hippocampus were measured. Microglia, insulin receptors, NLRP3, and IL‐1β in the hippocampus of mice were evaluated by immunofluorescence or immunohistochemical staining.

**Results:**

As anticipated, dapagliflozin improved insulin resistance and glucose metabolism and reduced cognitive impairment in female mice fed with a HFD. In the hippocampus, dapagliflozin alleviated microglial activation yet did not reduce the secretion of inflammatory chemokines. Furthermore, it increased the expression of insulin receptor in the hippocampus of HFD‐fed mice and decreased the expression of p‐tau.

**Conclusions:**

Our results provide a foundation for the clinical application of SGLT2is as an adjuvant to slow down the progression of central degenerative diseases related to metabolic disorders, such as AD.

## Graphical abstract

A graphic representation of the underlying mechanism of dapagliflozin improving high‐fat diet‐induced cognitive impairment in female mice.

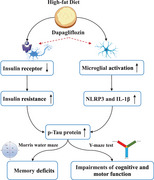

## 1 Introduction

Sodium‐glucose cotransporter 2 inhibitors (SGLT2is) are a novel group of antihyperglycemic drugs for treating type 2 diabetes mellitus (T2DM). Their main mechanism of action is to block glucose with SGLT2 in the proximal tubules of the kidney and inhibit urine glucose reabsorption (Nauck 2014). SGLT2 is also expressed in the hippocampus, hypothalamus, and blood–brain barrier (BBB) endothelial cells (X. Chen et al. 2024; Chiba et al. 2020). SGLT2is are lipid‐soluble drugs that can traverse the BBB and reach the central nervous system (CNS). Evidence suggests that SGLT2is have been shown to have anti‐inflammatory and neuroprotective effects in patients and animals with T2DM (Hayden et al. 2019; Hierro‐Bujalance et al. 2020; Khan et al. 2021).

Several studies have demonstrated that T2DM contributes to cognitive dysfunction, and insulin resistance (IR) in the hippocampus plays an important role in the cognitive decline associated with neuroinflammation and oxidative stress (Campillo et al. 2022; Wang et al. 2022). High‐fat diet (HFD)‐induced T2DM mouse models exhibit hyperglycemia, impaired glucose tolerance, hyperinsulinemia, hyperleptinemia, and hippocampal IR, particularly the downregulation of insulin receptor expression in the brain (J. M. Kim et al. 2020). Therefore, HFD‐induced obese mice are usually used to study the pathophysiological mechanisms linking IR and cognitive dysfunction (de Paula et al. 2021). Despite numerous publications on the impact of a HFD on the hippocampus in male rodents, few studies have evaluated this topic in female rodents.

Although the underlying pathophysiological mechanism of how a HFD affects hippocampal function remains unclear, it has been suggested that microglial activation and neuroinflammation play independent roles. Microglia are immune cells specific to the nervous system, and studies have indicated that they can have both beneficial and deleterious effects depending on their surroundings (Clark et al. 2021; Nguyen et al. 2020). HFD, which is rich in long‐chain saturated fatty acids (LCSFAs), can directly impact the CNS and promote microglial activation, leading to neuronal inflammation (J. D. Kim et al. 2019; Merlini et al. 2019; So et al. 2023). HFD intake has also been associated with cognitive and memory impairments, as well as an increase in phosphorylated tau (p‐tau) protein in the mouse hippocampus (Duffy et al. 2019; Elahi et al. 2021). Therefore, HFD has been used to establish experimental mouse models of T2DM and Alzheimer's disease (AD). Although the etiology of AD is not fully understood, evidence suggests that microglia‐mediated neuroinflammation contributes to its pathology and progression (Baik et al. 2019; Kang et al. 2021; Lin et al. 2021; Yang et al. 2022).

Given the role of a HFD in neuronal impairment and the anti‐inflammatory effects of SGLT2i administration, in this study, we aimed to investigate the impact of a long‐term HFD on cognitive function in female mice, as well as the potential for dapagliflozin to improve this impairment. We also sought to determine whether microglia‐mediated neuroinflammation is involved in this process.

## 2 Methods and Materials

### 2.1 Animals and Study Design

Four‐week‐old female C57BL/6J mice were obtained from the Experimental Animal Center. The study was approved by the Ethics Committee of Wuhan University, China (IRB approval number: WDRM 20210612) and followed ARRIVE guidelines 2.0. Mice were housed in a temperature‐controlled (22–23°C) and humidity‐controlled (45%–55%) room with a 12‐h light–dark cycle. All experiments using these mice were approved by the China Guidelines for the Care and Use of Experimental Animals. Mice were randomly divided into four groups: normal diet (Chow, *n* = 8) (D12450B, 385 kcal/100 g, 16% energy as fat), normal diet plus dapagliflozin (1 mg/kg) (Chow + Dapa, *n* = 8), HFD (*n* = 12), and HFD plus dapagliflozin (1 mg/kg) (HFD + Dapa, *n* = 12) (D12492, 524 kcal/100 g, 60% energy as fat). Dapagliflozin (5 mg/kg) was administered to mice in the diet (dosing according to a daily food intake of ∼5 g for mice) (Wei et al. 2020). One mouse in the normal diet group died due to improper operation by the breeder.

### 2.2 Behavioral Measurements

Behavioral testing was conducted at 27 weeks of age using the Morris water maze (MWM) test. The MWM consisted of a 120 cm diameter, 50 cm deep circular pool with opaque water maintained at 22.0 ±1°C. A 10 cm diameter platform was placed in the pool. The pool was divided evenly into four quadrants: N, S, E, and W (Figure 2a). The MWM is consisting of 6 days trials and included three parts: a visible platform period (1 day), a hidden platform period (4 days), and a probe trial (1 day) with a duration of 60 s. The platform was removed from the water during probe trial. All test procedures and data analysis methods are referred to in the literature (Bromley‐Brits et al. 2011).

The one‐trial Y‐maze test was performed 24 h after the probe trial. The Y‐maze consisted of three equal arms at a 120° angle, with each arm having internal dimensions of 35 cm × 5 cm × 15 cm high (Figure 3a). During the test, all three arms were opened, and each mouse was placed in the maze and allowed to freely explore for 10 min. The video system recorded the movement and path tracing. The six main outputs from the spontaneous alteration Y‐maze analysis are the number of entries, rotation, alternation, distance, mean speed, as well as percent alteration. Perfect alteration was defined as an animal entering all three arms in turn, that is, the ABC, BCA, or CBA. The percentage of perfect alternation is calculated as the ratio of the actual number of possible alternations to the maximum number of possible alternations.

### 2.3 Glucose and Insulin Tolerance Testing, and Body Composition

Intraperitoneal glucose tolerance tests (IPGTTs) and intraperitoneal insulin tolerance tests (IPITTs) were performed at 9 and 26 weeks of age, respectively. For the IPGTTs, a single dose of glucose (2 g/kg) was administered after 10 h of fasting, while for the IPITTs, a single dose of insulin (0.7 U/kg; Novo Nordisk) was given after 6 h of fasting. Blood sugar levels were measured using an FR201BC glucometer (glucose oxidase method) (MEDISAFE FIT, TERUMO, Japan). Body fat percentage and bone mineral density (BMD) were assessed in 26‐week‐old mice using dual‐energy x‐ray absorptiometry (Hologic, Discovery WI, USA).

### 2.4 ELISA for Cytokine and Hormone

The mice were deeply anesthetized with sodium pentobarbital (1.5%) after fasting for 6 h, and blood samples were collected at 28 weeks of age. Serum insulin and leptin levels were determined using an ultrasensitive mouse ELISA kit (Crystal Chem, USA). Serum interleukin 1β (IL‐1β) was measured using ELISA kits (MyBioSource, USA). The intra‐ and inter‐assay variations were 4.7–10.7 and 5.9–11.9, respectively.

### 2.5 Immunohistochemistry (IHC) and Immunofluorescence Staining

Mice were perfused with 0.9% saline solution followed by 4% paraformaldehyde (PFA) through the left cardiac ventricle. The brains were fixed in 4% PFA for 24 h at 4°C and then transferred to 30% sucrose and embedded in optimal cutting temperature (OCT) compound. Serial coronal sections (12 µm) of the brains were obtained using a cryostat (Leica Microsystems, CM1860). The brains were collected from the mice at the diestrus stage.

For immunohistochemical staining, frozen sections were washed with PBS and then incubated in blocking buffer at room temperature for 1 h. Next, the sections were incubated with primary antibodies (anti‐IL‐1β, 1:1000; anti‐NLRP3, 1:1000; anti‐insulin receptor, 1:1000; Servicebio, Wuhan, China; anti‐SGLT2 1:50; Proteintech Group Inc., Wuhan, China). Then, the sections were incubated with a secondary antibody (HRP‐conjugated anti‐rabbit immunoglobulin G, 1:200; Servicebio, Wuhan, China) at 37°C for 2 h. The slides were counterstained with 4′,6‐diamidino‐2‐phenylindole dihydrochloride (DAPI).

For immunofluorescence staining, frozen sections were washed with PBS and blocked with 3% bovine serum albumin for 30 min at room temperature. The slices were then incubated with primary antibodies, that is, anti‐Iba‐1(1:500), anti‐Neun (1:2000) (Servicebio, Wuhan, China), and anti‐SGLT2 (1:100), overnight at 4°C. After washing with PBS, the slices were incubated with a conjugated anti‐rabbit secondary antibody for 1 h at room temperature and then stained with DAPI dye. All the slices were digitally scanned using a Pannoramic 250 FLASH scanner (3DHISTECH, Hungary).

### 2.6 Quantification Method of Immunohistological Images

For quantitative analysis of microglia in the hippocampus, IBA‐positive cells (IBA^+^) with a visible cell body were counted in the dentate gyrus (DG) and the Cornu Ammonis Areas 1 and 3 (CA1 and CA3) regions from bregma −1.67 to bregma −1.91 mm. ImageJ plugin software was used to quantify the immunohistological images. An oval counting box or measurement window of 600 × 200, 300 × 500, 400 × 200 (μ m) was selected at the corresponding locations of CA1, CA3, and DG on the hippocampal images for microglia counting or immunohistochemical analysis. Five cells from each mouse were selected for analysis of the microglial soma area. The expression of the IL‐1β, NLRP3, and insulin receptor proteins was also quantified in the hippocampal DG, CA1, and CA3 regions. All counts and quantitative immunohistochemical analyses were conducted by blinded observers.

### 2.7 Statistics

All the data were analyzed using GraphPad Prism (v 9.0) with one‐way or two‐way analysis of variance (ANOVA), followed by Tukey's multiple comparison test. Differences were considered statistically significant at *p* < 0.05. The data are presented as the mean ± SEM.

## 3 Results

### 3.1 Metabolic Profile of HFD‐Fed Mice Treated With Dapagliflozin

HFD‐fed mice treated with dapagliflozin exhibited a significant decrease in fat mass and a delayed body weight gain. The daily food and water intake of dapagliflozin‐treated mice was greater than that of untreated mice. Notably, in dapagliflozin‐treated Chow‐fed mice, caloric intake was equal to that of HFD‐fed mice. This result suggested that dapagliflozin promoted both caloric intake and metabolism in mice, ultimately leading to improved weight gain. Moreover, it had no effect on lean body mass or BMD in Chow‐ or HFD‐fed mice (Table 1), which is consistent with the findings of clinical studies (Ha et al. 2021; Li et al. 2019). Furthermore, we examined the effects of dapagliflozin on glucose metabolism and insulin sensitivity. Compared with the Chow diet, HFD consumption increased serum glucose levels, impaired glucose tolerance, and increased the area under the curve (AUC); however, these effects were partially ameliorated by dapagliflozin treatment. To examine its effect on insulin sensitivity, all mice were subjected to the IPITTs. Fasting glucose levels were similar in both Chow‐ and HFD‐fed mice. Blood sugar levels were significantly greater in HFD‐fed mice than in Chow‐fed mice at 15 and 30 min after insulin injection. However, 45 min after injection, most mice, both Chow‐ and HFD‐fed mice, showed hypoglycemia. Conversely, mice treated with dapagliflozin showed normal and steady plasma glucose levels. The AUC did not significantly differ among the four groups (Table 2).

**TABLE 1:** Metabolic parameters: data are presented as mean ± SEM (*n* = 7–12 per group).

Group	Chow	Chow + Dapa	HFD	HFD + Dapa
Weight (4 weeks, g)	15.14±0.13	15.77±0.28	14.64±0.13	14.62±0.16
Weight (28 weeks, g)	20.36±0.25 ^####^	21.60±0.23 ^#^	26.81±0.70∗∗∗∗	22.66±0.34 ^ΔΔΔΔ^
Diet (g/d)	2.13±0.08	2.42±0.09 ^¥^	1.75±0.04**	1.92±0.03 ^ΔΔ^
Diet (kcal/d)	8.19±0.31	9.30±0.34 ^¥^	9.16±0.20*	10.07±0.16 ^ΔΔ^
Drinking (mL/d)	4.52±0.10	5.26±0.17 ^¥¥^	3.51±0.09∗∗∗∗	4.34±0.12 ^ΔΔΔΔ##^
Lean mass (g)	18.45±0.40**^##^	19.63±0.48	20.97±0.24	19.64±0.32
Fat mass (g)	2.82±0.28**	2.58±0.36**	7.50±0.59	3.63±0.43 ^Δ^
Fat %	13.24±1.29****	11.69±1.68****	27.10±1.58	15.17±1.58 ^ΔΔΔΔ^
BMD (g/cm^2^)	0.099±0.007	0.097±0.007	0.096±0.004	0.100±0.004
Subcutaneous fat (g)	0.106±0.010****^##^	0.089±0.006***^###^	0.303±0.034	0.151±0.010^ΔΔ^
Subperitoneal fat (g)	0.321±0.028	0.304±0.025	0.847±0.085***^###^	0.531±0.040^Δ^
Gonadal fat (g)	0.026±0.003***	0.022±0.003****^#^	0.047±0.004	0.023±0.002^ΔΔΔ^

*Note*: Data are analyzed by one‐way ANOVA and Tukey's multiple comparison test: *p < 0.05, **p < 0.01, ***p < 0.001, ****p < 0.0001 HFD vs. Chow or Chow + Dapa. ^#^ *p* < 0.05, ^##^ *p* < 0.01, ^###^ *p* < 0.001, ^####^ *p* < 0.0001 HFD + Dapa vs. Chow or Chow + Dapa. ^Δ^ *p* < 0.05, ^ΔΔ^ *p* < 0.01, ^ΔΔΔ^ *p* < 0.001, ^ΔΔΔΔ^ *p* < 0.0001 HFD vs. HFD + Dapa. ^¥^ *p* < 0.05, ^¥¥^ *p* < 0.01, Chow vs. Chow + Dapa.

**TABLE 2:** IPGTTs and IPITTs: data are presented as mean ± SEM (*n* = 7–12 per group).

Time∖Group		Chow	Chow + Dapa	HFD	HFD + Dapa
IPGTT (mmol/L)	0 (min)	5.47±0.25	5.45±0.37	7.92±0.47	6.13±0.59
9‐week	30	8.40±0.45 *####*	9.40±2.21 ^###^	15.68±1.67****	12.79±0.89 ^Δ^
	60	7.25±0.37	7.10±0.68	8.62±0.46	9.18±0.58
	90	6.73±0.25	7.01±0.50	7.48±0.34	8.30±0.66
	120	5.58±0.46	5.31±0.48	4.90±0.84	8.56±0.44
AUC_glucose_ (mmol/L × min/L)		27.91±1.27*	31.27±4.81*	38.19±2.98	37.70±2.71
26‐week	0 (min)	6.57±0.93	5.97±0.80	7.95±1.56	7.41±1.25
	30	10.82±2.53 ^####^	10.22±1.21 ^####^	20.71±4.56****	16.32±3.52 ^ΔΔΔΔ^
	60	8.53±1.29	8.61±1.06	12.47±2.64****	11.41±2.54 ^##^
	90	7.50±1.17 *^#^*	7.97±1.04	10.23±1.94*	9.87±2.00
	120	7.26±1.09	7.36±0.74	8.96±1.69	9.19±1.77
AUC_glucose_ (mmol/L × min/L)		33.77±2.29	33.65 ± 1.46	51.86 ±4.13**	45.90 ± 3.55
IPITT (mmol/L)	0	7.67±0.56	7.64±1.59	7.86±0.78	8.34±1.12
26‐week	15	4.28±1.45	4.00±1.02	5.30±1.09	4.82±0.92
	30	2.40±0.79 ^#^	4.12±1.21 ^¥¥^	3.38±0.94	3.71±0.95
	45	1.77±0.79^####^	3.63±1.25^¥¥^*	2.29±1.27	4.01±0.91^ΔΔ^
AUC_glucose_ (mmol/L × min/L)		11.39 ± 1.27	17.49 ± 1.85	13.75 ±1.26	18.73 ± 1.41

Note: *p < 0.05, **p < 0.01, ****p < 0.0001 HFD vs. Chow or Chow + Dapa. #*p* < 0.05, ##*p* < 0.01, ###*p* < 0.001, ####*p* < 0.0001 HFD + Dapa vs. Chow or Chow + Dapa. Δ*p* < 0.05, ΔΔ*p* < 0.01, ΔΔΔΔ*p* < 0.0001 HFD vs. HFD + Dapa. ¥¥*p* < 0.01, Chow vs. Chow + Dapa.

### 3.2 Dapagliflozin and HFD Induce Changes in Serum Biochemical

**FIGURE 1:**
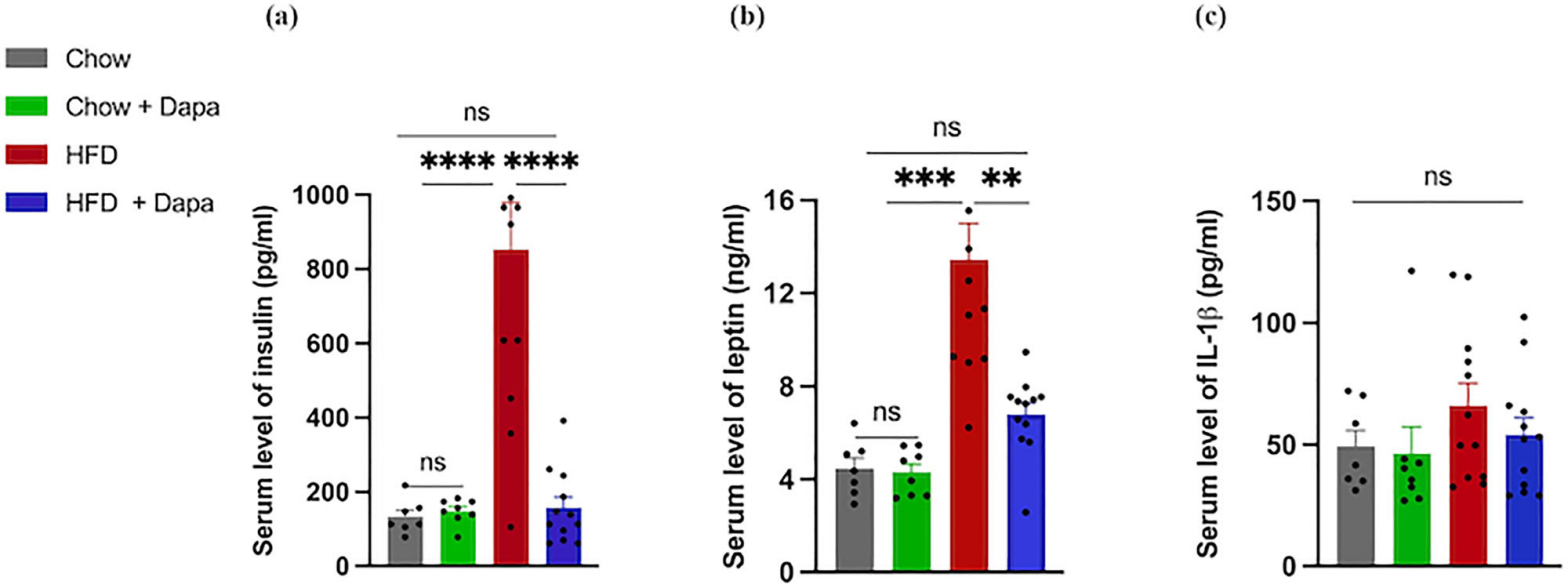
Effects of HFD and dapagliflozin on the serum levels of insulin, leptin, and inflammatory factors (*n* = 7–12 mice per group, one‐way ANOVA followed by Tukey's multiple comparisons test). (a) Insulin, (b) leptin, and (c) IL‐1β. The data are the means ± SEM. One‐way ANOVA: **p < 0.01, ***p < 0.001, ****p < 0.0001 for the HFD group versus the Chow, Chow + Dapa, or HFD + Dapa groups.

Changes in fat mass are often linked to changes in serum insulin and leptin levels (Friedman 2019). In our study, we observed significantly increased insulin and leptin levels in the serum of HFD‐fed female mice, whereas HFD‐fed mice treated with dapagliflozin exhibited significantly decreased serum insulin and leptin levels (Figure 1a,b). Although the body weight of dapagliflozin‐treated HFD‐fed mice was greater than that of Chow‐fed mice, there was no significant difference in insulin or leptin levels among the three groups of mice. This finding suggested that dapagliflozin improved insulin and leptin sensitivity, which was partly independent of body weight gain. Previous studies have reported an association between brain IR‐induced neuroinflammation and cognitive decline (Esmaeili et al. 2020; Feng et al. 2017); therefore, we measured the expression of IL‐1β in both the serum and hippocampus. The results showed that the serum IL‐1β levels were greater in HFD‐fed mice than in Chow‐fed mice, although this difference was not statistically significant (Figure 1c). The results for IL‐1β in the hippocampus are described in the following sections.

### 3.3 Dapagliflozin Attenuates the HFD‐Induced Cognitive Impairment

**FIGURE 2:**
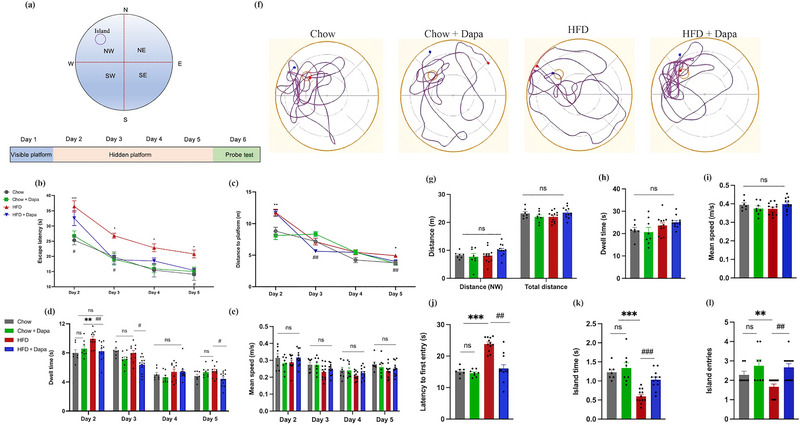
Results of the analysis of different parameters in mice during the MWM test (*n* = 7–12 mice per group, two‐way ANOVA followed by Tukey's multiple comparisons test). (a) Four equally sized quadrants in the MWM pool, the hidden platform period; (b) escape latency; (c) distance to platform; (d) dwell time; (e) mean speed, the probe trial; (f) representative track images of four groups of mice during the probe trial; (g) distance in the target quadrant and total paths; (h) dwell time; (i) mean speed; (j) latency to first entry; (k) island time; and (l) island entries. The data are the means ± SEM. *p < 0.05, **p < 0.01, ***p < 0.001 HFD versus Chow or Chow + Dapa; *^#^p* < 0.05, *^##^p* < 0.01, *^###^p* < 0.001 HFD versus HFD + Dapa.

To investigate the effects of dapagliflozin on cognitive function, the MWM and Y‐maze tests were conducted. The MWM is commonly used to evaluate long‐term learning and memory abilities, with the test during the hidden platform period (Days 2–5) considered as the acquisition trial. The escape latency, distance to the platform, dwell time, and mean speed were analyzed daily. Figure 2f shows track images of the mice in the probe trial.

First, there was not any difference among groups on Day 1 (results not shown), indicating that all groups have similar motor and visual capabilities. For Day 2–5, all groups of mice showed a significant decrease in escape latency. Two‐way ANOVA within each day revealed that HFD‐fed mice had the longest escape latency compared to the other three groups, and dapagliflozin‐treated mice demonstrated a significant decrease in escape latency on the third and fifth days, suggesting a potential benefit in attenuating HFD‐induced cognitive impairment (Figure 2b). Second, all groups of mice showed a significant decrease in distance to the platform from Days 2 to 5. Two‐way ANOVA within each day showed that the HFD‐fed mice swam longer distances to find the hidden platform than did the Chow‐fed control mice on the second day and fifth day, and HFD‐fed mice treated with dapagliflozin showed a significant decrease in distance to the platform on Days 3 and 5 (Figure 2c). The finding suggested that dapagliflozin effectively improves learning and memory deficits induced by a HFD.

However, the differences in dwell time in the platform quadrant among the four groups were puzzling. There was a significant increase in HFD‐fed mice compared to Chow‐fed mice. Further analysis revealed that compared with untreated HFD‐fed mice, HFD‐fed mice treated with dapagliflozin showed a significant reduction in dwell time on the second, third, and fifth days (Figure 2d), possibly related to escape latency. Additionally, there was no difference in the mean speed among the four groups, implying that the cognitive dysfunction induced by HFD was independent of motor incoordination (Figure 2e).

To assess the impact of a HFD on cognition and the benefits of dapagliflozin, a probe trial was performed on the sixth day. The distance traveled, dwell time, mean speed, latency to first entry, island time, and number of entries were recorded and analyzed. Figure 2f shows track images from the probe trial. There was no statistical difference in distance, dwell time, or mean speed among the four groups (Figure 2g–i). However, in contrast to the other three groups, the latency to first entry was prolonged in the HFD group, and the island time and number of island entries were reduced, which was significantly improved after treatment with dapagliflozin (Figure 2j–l). These data indicated that dapagliflozin treatment significantly improved the memory deficits seen in HFD‐fed mice.

**FIGURE 3:**
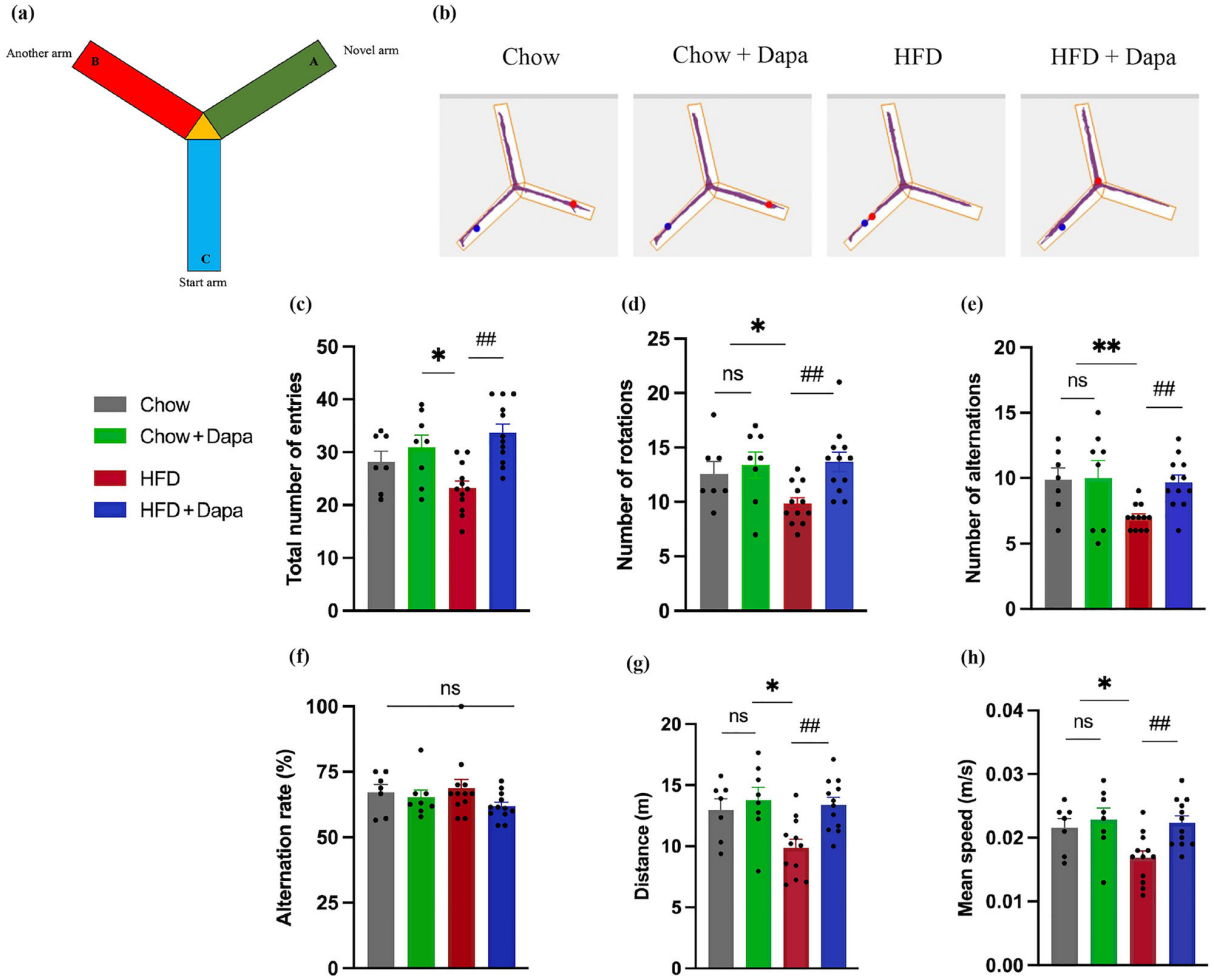
The results of different analysis parameters for mice during the Y‐maze test and illustrated animal track images (*n* = 7–12 mice per group, one‐way ANOVA followed by multiple unpaired *t*‐tests). (a) Y‐maze apparatus, (b) representative track images of four groups of mice, (c) total number of entries, (d) number of rotations, (e) number of alternations, (f) alternation rate (%), (g) distance during 10‐min test, and (h) mean speed. The data are the means ± SEM. *p < 0.05 HFD versus Chow or Chow + Dapa; *^#^p* < 0.05, *^##^p* < 0.01 HFD versus HFD + Dapa.

The Y‐maze test is widely used to evaluate the cognitive and motor functions in the mice. The total number of arm entries and the frequency of alternations were recorded to assess the spatial memory and exploratory behavior of the mice. The number of rotations, walking distance, and movement speed reflect the mouse's motor coordination and agility. Figure 3b shows the track images of the mice in the test. As shown in Figure 3c–e, one‐way ANOVA revealed that HFD‐fed mice had fewer total arm entries, rotation, and alterations than did the other groups, with improvements after dapagliflozin treatment. There were no significant differences in the alteration rates among the groups (Figure 3f). The total distance traveled by HFD‐fed mice was shorter than that traveled by Chow‐fed mice but increased significantly with dapagliflozin treatment (Figure 3g). The mean speed of the HFD group mice decreased compared to that of the control group mice but improved with dapagliflozin treatment (Figure 3h). These results indicated that dapagliflozin treatment ameliorates the impairments of cognitive and motor functions caused by HFD.

### 3.4 Dapagliflozin Attenuates Hippocampal Microglial Activation in HFD‐Fed Mice

**FIGURE 4:**
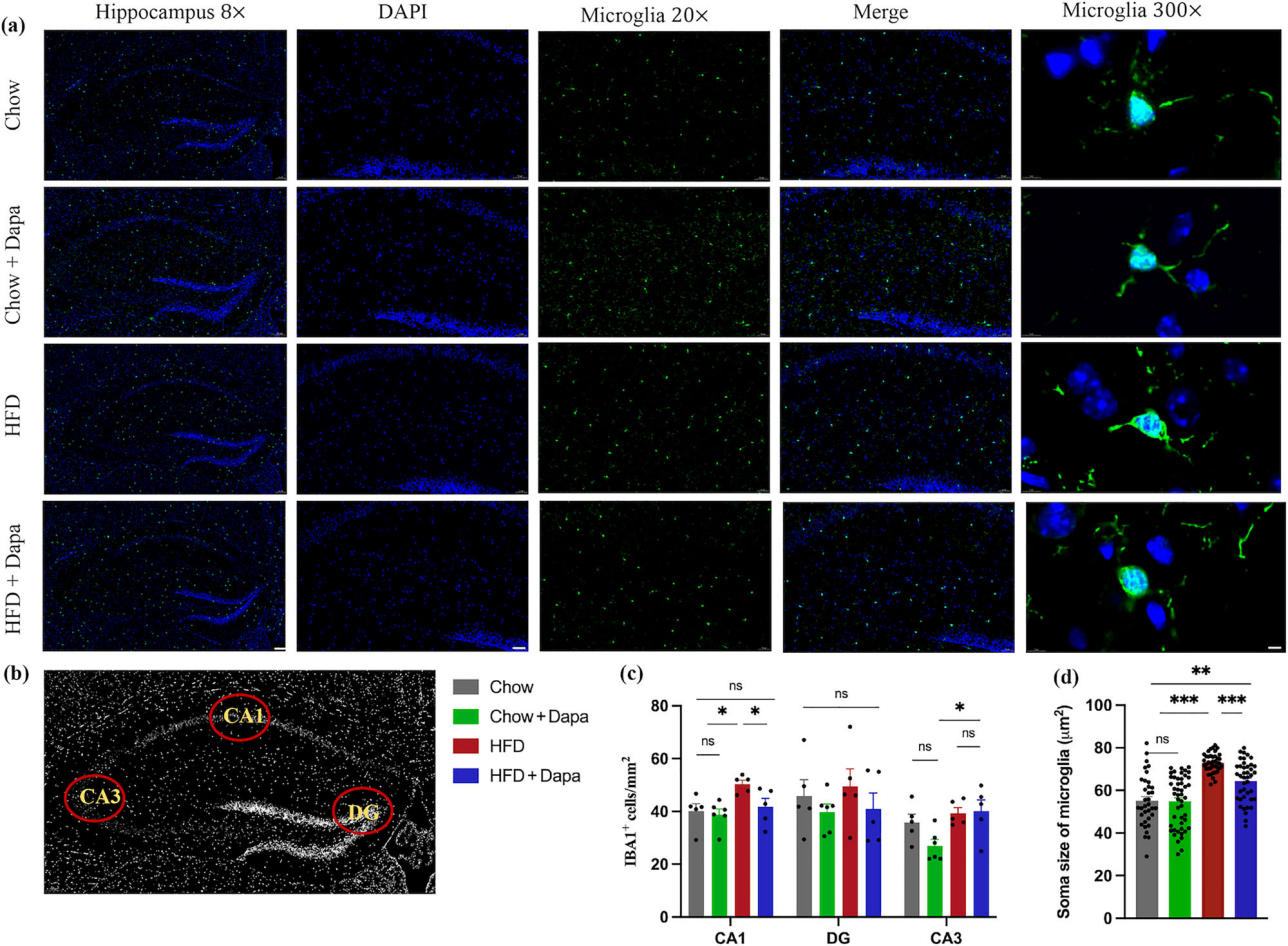
Effects of HFD and dapagliflozin on hippocampal microglial activation (*n* = 4–6 mice per group, two‐way ANOVA followed by Tukey's multiple comparisons test). (a) Representative immunofluorescence images of IBA1‐labeling cells counterstained with DAPI (scale bar = 100, 50, or 5 µm). (b) Representative image of the localization of IBA1 in the hippocampal DG, CA1, and CA3; (c) quantification of IBA1^+^ cells in the hippocampus; and (d) soma size of IBA1^+^ cells. The data are the mean s±SEM. *p < 0.05, ***p < 0.001, HFD versus Chow or Chow + Dapa or HFD + Dapa; **p < 0.01, HFD + Dapa versus Chow or Chow + Dapa.

The number and size of microglia in the hippocampal DG, CA1, and CA3 were analyzed by immunostaining (Figure 4a,b). The results showed that HFD‐fed mice had significantly more microglia in the hippocampal CA1 and CA3 regions than Chow‐fed mice (Figure 4c). Treatment with dapagliflozin led to a decrease in the number of microglia in the hippocampal CA1 region, indicating the potential of dapagliflozin to inhibit HFD‐induced microglial priming. Additionally, the soma size of microglia was larger in HFD‐fed mice than in both Chow‐fed and dapagliflozin‐treated HFD‐fed mice, illustrating HFD‐related morphological changes in microglia (Figure 4d).

**FIGURE 5:**
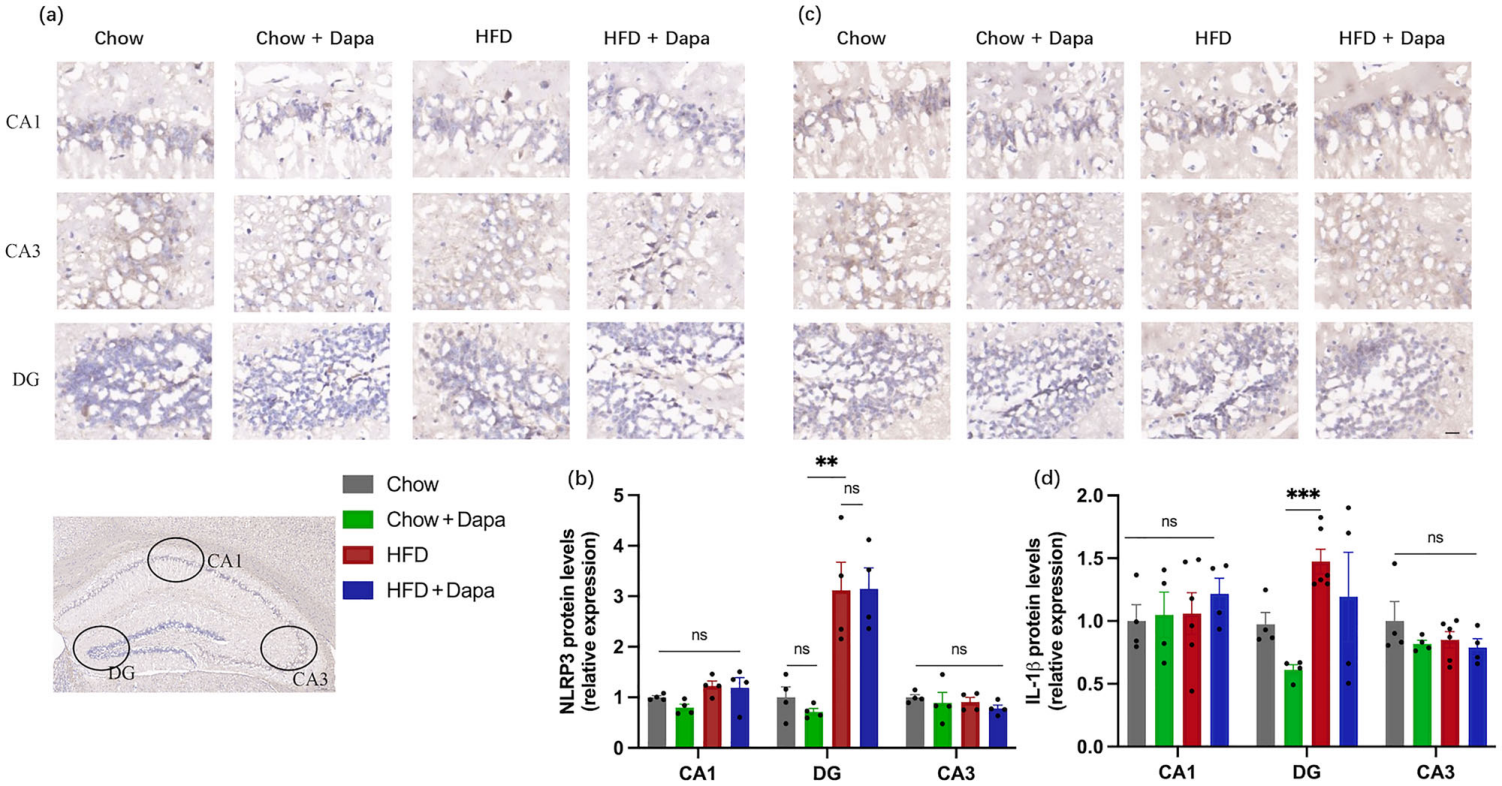
Effects of HFD and dapagliflozin on the hippocampal expression of NLRP3 and IL‐1β in the hippocampus (*n* = 4–6 mice per group, two‐way ANOVA followed by Tukey's multiple comparisons test). (a) Representative immunohistochemistry images of NLRP3, (b) quantitative analysis of the protein expression of NLRP3, (c) representative immunohistochemistry images of IL‐1β, and (d) quantitative analysis of the protein expression of IL‐1β (scale bar = 50 µm). The data are the means ± SEM. **p < 0.01, ***p < 0.001, HFD versus Chow or Chow + Dapa.

The levels of NLRP3 in the hippocampal DG, CA1, and CA3 were evaluated using immunohistochemical staining (Figure 5a). Both HFD‐fed mice and HFD‐fed mice treated with dapagliflozin showed significantly greater levels of NLRP3 than Chow‐fed and dapagliflozin‐treated Chow‐fed mice (Figure 5b). The expression of IL‐1β in the hippocampal DG, CA1, and CA3 regions is shown in Figure 5c. The hippocampal DG was more highly expressed in HFD‐fed mice than in dapagliflozin‐treated Chow‐fed mice (Figure 5d).

Taken together, these findings demonstrate that long‐term consumption of a HFD may lead to hippocampal inflammation through microglial activation, which is not prevented by dapagliflozin.

### 3.5 Dapagliflozin Attenuates Hippocampal Insulin Resistance in HFD‐Fed Mice

**FIGURE 6:**
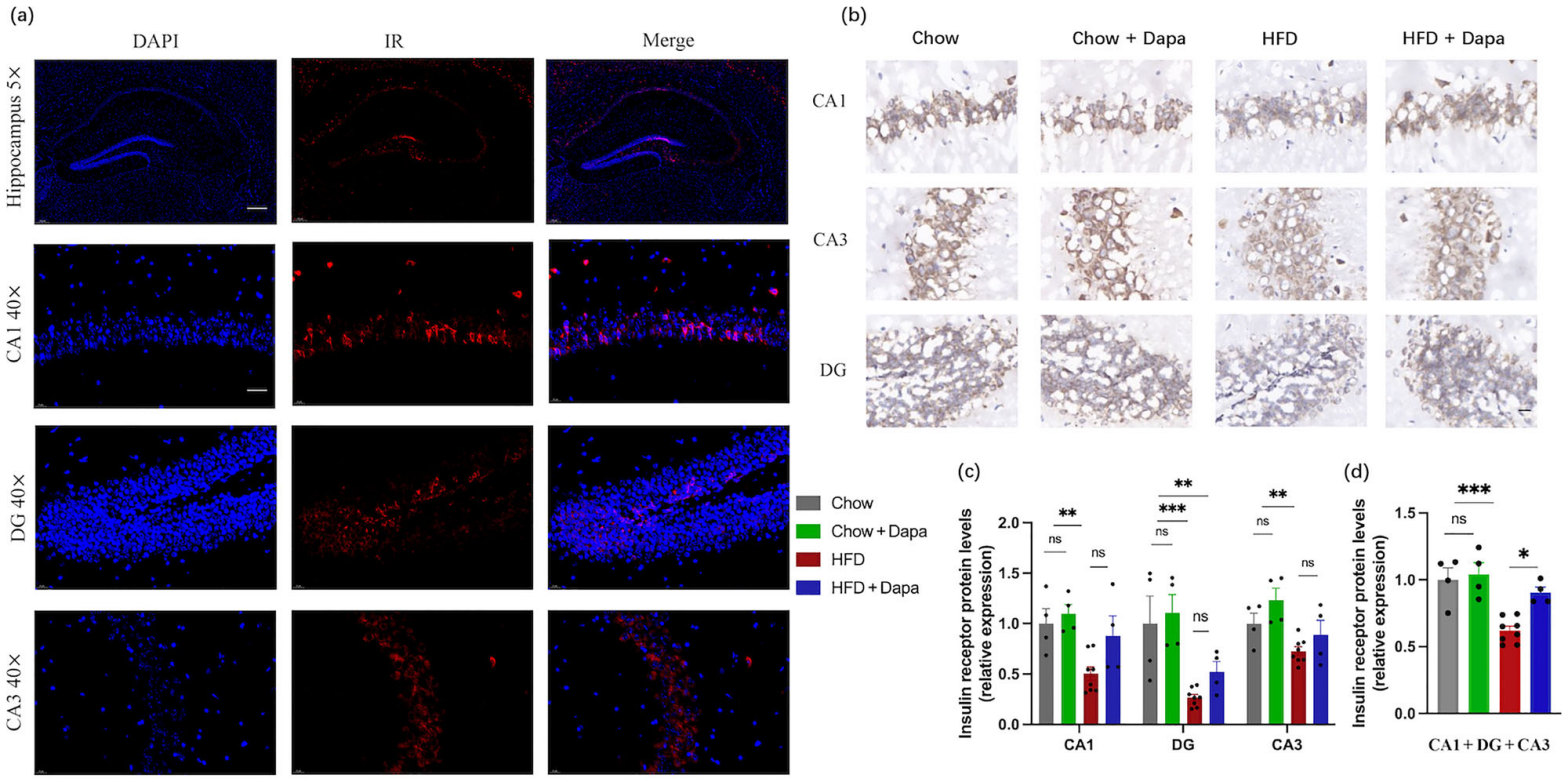
Effects of HFD and dapagliflozin on hippocampal expression of insulin receptors in the hippocampus (*n* = 4–8 mice per group, two‐way ANOVA followed by Tukey's multiple comparisons test). (a) Representative immunohistochemistry image of the insulin receptor (scale bar = 200 and 20 µm), (b) representative immunohistochemistry images of the insulin receptor (scale bar = 50 µm), and (c, d) quantitative analysis of the insulin receptor protein. The data are the means ± SEM. *p < 0.05, HFD versus HFD + Dapa; **p < 0.01, ***p < 0.001, HFD versus Chow or Chow + Dapa.

The brain, especially the hippocampus, is sensitive to insulin (Milstein and Ferris 2021). Figure 6a shows the expression of insulin receptors (IRs) in the hippocampus. The expression of IRs in the hippocampal CA1, DG, and CA3 regions was lower in HFD‐fed mice than in Chow‐fed mice (Figure 6b,c). Dapagliflozin treatment partly improved this expression (Figure 6d). Unfortunately, we did not evaluate the signaling molecules downstream of the insulin receptor in our study. Additionally, there was very little expression of the leptin receptor (LR) in the hippocampus, as observed through immunohistochemical staining and further verified by immunofluorescence staining (data not shown).

### 3.6 Expression of SGLT2 and Phosphorylated‐Tau in the Hippocampus of Mice

**FIGURE 7:**
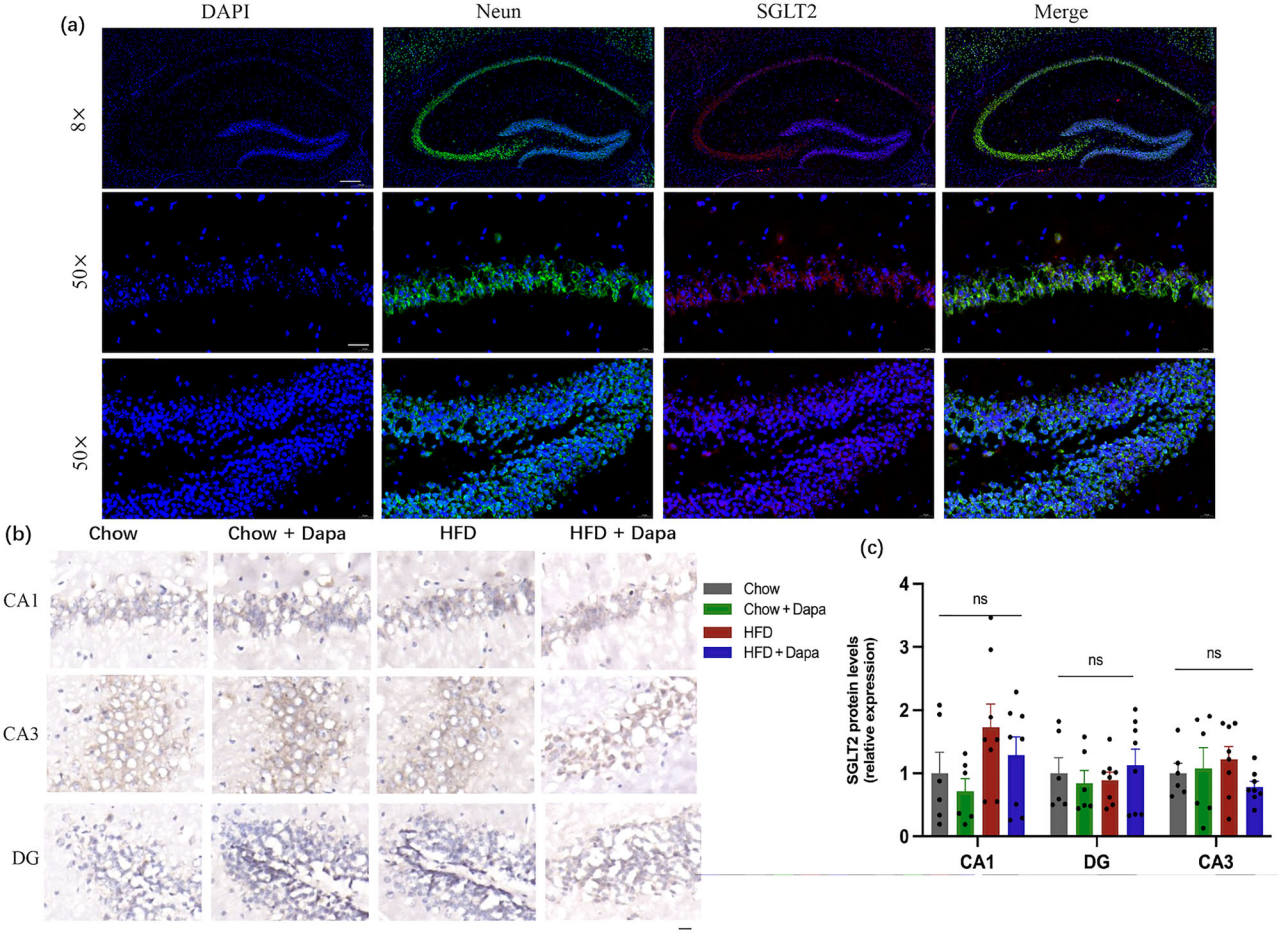
Expression of SGLT2 in the hippocampal DG, CA1, and CA3, and effects of dapagliflozin on the expression of SGLT2 (*n* = 6 mice per group, two‐way ANOVA followed by Tukey's multiple comparisons test). (a) Immunofluorescence images of Neun and SGLT2 labeling (scale bar = 200 or 20 µm), (b) representative immunohistochemistry images of SGLT2 (scale bar = 50 µm), and (c) quantitative analysis of SGLT2 protein expression. The data are the means ± SEM.

Digital brain slice images labeled with NeuN (green)/SGLT2 (red) showed SGLT2 presence in the mouse hippocampal DG, CA1, and CA3 (Figure 7a). SGLT2 expression in the hippocampus did not significantly differ among the four groups of mice (Figure 7b,c). This result may be verified using other methods.

**FIGURE 8:**
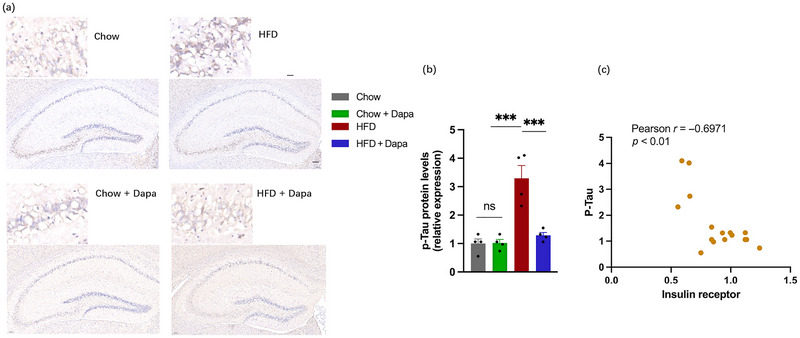
Expression of p‐tau in the hippocampus and effects of dapagliflozin on the expression of p‐tau (*n* = 4 mice per group, two‐way ANOVA followed by Tukey's multiple comparisons test). (a) Representative immunohistochemistry images of p‐tau (scale bar = 50 or 100 µm), (b) quantitative analysis of the protein of p‐tau protein, and (c) correlation between the insulin receptor and p‐tau. The data are the means ± SEM. ***p < 0.001, HFD versus Chow, Chow + Dapa, or HFD + Dapa.

To investigate the neuroprotective effects of dapagliflozin on HFD‐induced cognitive dysfunction, phosphorylated tau (p‐tau) protein levels in the hippocampus were evaluated using immunohistochemical staining (Figure 8a). One‐way ANOVA showed that the p‐tau protein levels in HFD‐fed mice were greater than that in the other groups (Figure 8b). Correlation analysis revealed a negative correlation between insulin receptor expression and p‐tau expression (Figure 8c).

## 4 Discussion

Previous studies have demonstrated that long‐term consumption of a HFD not only leads to increased body weight and metabolic imbalance but also causes deficits in recognition and spatial memory through neuroinflammation (Hernandez‐Ramirez et al. 2022; J. M. Kim et al. 2020; Pan et al. 2023). In our recent study, we noted an elevated expression of inflammatory cytokines NLRP3 and IL‐1β in the DG region of the hippocampus and microglial activation in female mice fed a HFD for 24 weeks, which contradicts previous reports that did not find hypothalamic inflammation in female mice (K. Chen et al. 2021; Lainez et al. 2018). This discrepancy may be due to the duration of the HFD feeding. Published researches have demonstrated that saturated fatty acids (SFAs) from a HFD, acting as common damage‐associated molecular patterns (DAMPs), have the ability to directly enter the brain. Upon binding to pattern recognition receptors (PRRs) such as CD36, they are taken up by microglia and activate it, leading to the promotion of synthesis and secretion of pro‐inflammatory molecules like IL‐1β and TNF‐α. It has become evident that the function of microglia is closely intertwined with energy metabolism. Microglia have the ability to undergo “metabolic reprogramming,” adjusting their internal metabolic pathways in different activation states (Gu et al. 2021; Kalambogias et al. 2020). Concurrently, as metabolic reprogramming toward glycolysis intensifies, the microglia phenotype shifts toward a pro‐inflammatory type. Furthermore, Milanova et al. (2019) stated that in a high‐lipid environment, the glucose utilization and oxidation of microglia declined, while the utilization of lipid substrates increased. Of course, these results require further verification through future experiments.

Although SGLT2is have been demonstrated to have beneficial effects on neurodegenerative diseases in rodent models (Hayden et al. 2019; Hierro‐Bujalance et al. 2020; Ibrahim et al. 2022; Khan et al. 2021; Rizzo et al. 2022), their role in the hippocampus of insulin‐resistant female models has not been addressed, and there is no study that has analyzed the effects of SGLT2i on cognitive impairment. Therefore, the aim of our study was to evaluate whether dapagliflozin, an SGLT2 inhibitor, could improve cognitive impairment caused by long‐term HFD consumption in female mice and to explore the underlying mechanisms involved.

In our study, mice showed a significant increase in body weight after being fed a HFD for 24 weeks. During the first 3 weeks of the experiment, the daily food, caloric, and water intake was significantly greater in mice treated with dapagliflozin than in nontreated mice, indicating the appetite‐promoting effects of dapagliflozin. Subsequently, mice fed Chow supplemented with dapagliflozin had body weight similar to those of Chow‐fed mice. Mice fed a HFD with dapagliflozin exhibited lower body weights than those fed a HFD alone. The results of body composition analysis showed that a HFD significantly increased lean and fat mass. However, only fat mass, including subcutaneous fat, subperitoneal fat, and gonadal fat, significantly decreased in mice fed a HFD with dapagliflozin treatment. There was no difference in BMD among the four groups of mice, suggesting that SGLT2i treatment had no adverse effects on bone metabolism. Our study is the first to propose that the positive impact of SGLT2i on cognitive function in HFD‐fed mice is linked to a reduction in white fat mass. Blood glucose levels were tested, and it was found that dapagliflozin did not significantly decrease fasting blood sugar levels in HFD‐fed mice. The results of the IPGTT demonstrated that dapagliflozin significantly improved glucose tolerance at 30 min after glucose injection; however, no significant difference was observed at 60, 90, or 120 min.

The consumption of a HFD significantly increased serum insulin and leptin levels but not IL‐1β levels. Dapagliflozin caused a dramatic decrease in serum insulin and leptin levels in HFD‐fed mice. Our findings suggest that after 24 weeks of HFD feeding, female mice do not exhibit systemic inflammation in the periphery. Future studies should assess different inflammatory markers such as IL‐6 and TNF‐α to evaluate inflammation status. Hyperinsulinemia has been shown to promote the release of proinflammatory factors from macrophages in adipose tissue (Puschel et al. 2022; Ying et al. 2021). Our results differ from studies conducted in male mice, where HFD feeding induced proinflammatory conditions after 4 weeks (Z. Wu et al. 2021). This may be due to the time point studied not reaching the peak of IL‐1β release or the anti‐inflammatory effect of estrogen in female mice.

In the present study, mice fed a HFD demonstrated an increase in the time taken to reach the platform during both the acquisition trial and the probe trial. Additionally, there was a decrease in the time spent in the island quadrant and in the number of crossings the island. These findings indicate deficits in learning and spatial memory functions in HFD‐fed mice. To better illustrate the mechanism underlying the beneficial effect of dapagliflozin on cognitive function, we investigated the expression of microglia and inflammatory factors in the hippocampus. Microglial activation plays an important role in obesity‐related neuroinflammation (Cope et al. 2018). In line with previous studies, our study confirmed that a HFD induced microglial activation and the production of NLRP‐3 and IL‐1β in the hippocampus (Mazzei et al. 2021; Medrano‐Jimenez et al. 2019; M. Wu et al. 2022). Importantly, our study revealed for the first time that dapagliflozin attenuated microglial activation in female mice, but did not reduce NLRP3 or IL‐1β expression. These results also suggest that the beneficial effects of dapagliflozin may not be associated with improvements in neuroinflammation.

Numerous studies have shown the significant role of insulin in various brain functions, and IR is an independent risk factors for cognitive impairment and AD (A. Kim and Arvanitakis 2023; Liu et al. 2022). IR‐induced hyperinsulinemia is also an independent risk factor for neuroinflammation in the CNS (Peng et al. 2021; Yang et al. 2022; Yin et al. 2020). Insulin, which is secreted from the pancreas, enters the circulation and crosses the BBB to act on all cell types in the brain via IRs (Milstein and Ferris 2021). IRs are expressed in neurons and glial cells and play a crucial role in determining cellular metabolism and functional status (Kumar et al. 2021). It has been recognized that the hippocampal DG, CA1, and CA3 play important roles in regulating learning and spatial memory ability (Hoge and Kesner 2007). Our data showed that HFD‐fed mice exhibited defects in IR expression in the hippocampus, as assessed by histopathological analysis via immunohistochemical staining. Dapagliflozin treatment significantly protected against HFD‐induced decreases in hippocampal IRs. In addition, we observed very low expression of LRs in the hippocampus. These results suggest that hyperinsulinemia and IRs are associated with cognitive dysfunction rather than hyperleptinemia and LRs. SGLT2 is a glucose transporter in brain cells. In our previous study, we confirmed SGLT2 expression in the hippocampus, hypothalamus, and BBB endothelial cells (X. Chen et al. 2024; Chiba et al. 2020). Therefore, it was expected that HFD feeding or dapagliflozin treatment would impact SGLT2 expression levels within the hippocampus. However, quantitative analysis of the immunohistochemical staining showed no significant difference. This result demonstrated that beneficial effects of dapagliflozin were not associated with SGLT2 expression in hippocampus. Yan et al. reported that adipose tissue‐derived EVs from HFD‐fed mice induced remarkable synaptic loss and cognitive impairment (Wang et al. 2022). In another study of ours, we also discovered that dapagliflozin alleviated adipocyte hypertrophy and insulin resistance caused by a HFD. Therefore, we hypothesize that the mechanism through which the inhibition of SGLT2 expression enhances cognitive function in HFD mice might be related to the reduction of vesicles secreted by adipose tissue.

In the present study, the expression of p‐tau protein in the hippocampus of HFD‐fed mice was increased, which was in accordance with Kim's findings, and the expression of p‐tau protein was negatively correlated with IRs expression (You et al. 2020). Furthermore, inhibiting SGLT2 resulted in the downregulation of p‐tau protein levels and upregulation of IRs (Ibrahim et al. 2022).

## 5 Conclusion

Overall, the present study suggested that hippocampal IR is the primary mechanism underlying cognitive impairment caused by long‐term HFD consumption in female mice. However, our results do not reveal whether hyperinsulinemia promotes microglial activation or whether neuroinflammation induced by microglial M1 polarization decreases the expression of insulin receptors and leads to insulin resistance in the hippocampus. Furthermore, considering its effects on hyperglycemia, neuroinflammation, and neuronal protection related to cognitive impairment, dapagliflozin is more likely to attenuate HFD‐induced cognitive decline by improving IR in the hippocampus. Thus, SGLT2i could be a promising therapeutic agent to prevent the development of cognitive decline in T2D patients. Future studies will focus on the relationship between SGLT2 and hippocampal IR, as well as that between peripheral adipose tissue and central neuronal function, in order to develop effective management strategies for cognitive decline in patients with T2DM.

## Author Contributions

**Xiaolin Chen**: conceptualization, investigation, funding acquisition, writing–original draft, methodology, writing–review and editing, formal analysis, supervision, software. **Mingxia Fan**: methodology, supervision, data curation. **Zhuoni Xiao**: validation, visualization, supervision, project administration. **Xiaoxing Xiong**: conceptualization, supervision, writing–review and editing, resources.

## Conflicts of Interest

The authors declare no conflicts of interest.

### Peer Review

The peer review history for this article is available at https://publons.com/publon/10.1002/brb3.70361

## Data Availability

Data will be made available on request.
